# Sustainable Extraction of Bioactive Phenolic Compounds from Cannabis Leaf Powder from Local Strain of Tucumán, Argentina, to Promote a Circular Economy

**DOI:** 10.3390/molecules31111845

**Published:** 2026-05-27

**Authors:** Bárbara Salinas Orellana, Fatima Carolina Danert, Iris Catiana Zampini, María Inés Isla

**Affiliations:** 1 Instituto de Bioprospección y Fisiología Vegetal (INBIOFIV), CONICET-Universidad Nacional de Tucumán (UNT), San Martín 1545, San Miguel de Tucumán T4000CBG, Tucumán, Argentina; barsalinas6@gmail.com (B.S.O.); fcdanert@gmail.com (F.C.D.); zampini@csnat.unt.edu.ar (I.C.Z.); 2 Facultad de Ciencias Naturales e IML, UNT, Miguel Lillo 205, San Miguel de Tucumán T4000CBG, Tucumán, Argentina

**Keywords:** *Cannabis sativa*, leaves, polyphenolic compounds, antioxidant, cannabinoids, ultrasonic assisted extraction, response surface methodology (RMS)

## Abstract

This study aimed to optimize the extraction of total phenolic compounds from *Cannabis sativa* L. leaf powder cultivated in Tucumán, Argentina (INBIOFIV 00500 Tuc chemotype), using ultrasound-assisted extraction (UAE), and to evaluate the process at a screening level in terms of environmental performance. Although leaves are not the primary raw material in the medicinal cannabis industry—where inflorescences are preferentially used due to their cannabinoid content—they represent an underutilized biomass with potential for valorization as a source of bioactive phenolic compounds for cosmetic, food, and pharmaceutical applications. The leaves were dried and ground to a particle size between 74 and 840 µm. The effects of process parameters, including solid-to-liquid ratio, solvent composition, and extraction time, were evaluated using response surface methodology (RSM). The optimal conditions predicted by the model were 46% ethanol, a solid-to-liquid ratio of 1:10 (*w*/*v*), 70% amplitude, and 30 °C. Although the model indicated a short optimal sonication time (1 min), experimental results showed that a plateau in extraction yield was reached at 6 min, with no significant increase thereafter. Under these conditions, the extract exhibited high total phenolic content (1746.83 µg GAE/mL) and total flavonoids (858.41 µg QE/mL), along with strong antioxidant activity. The extract showed no significant toxicity in the *Artemia salina* assay. The environmental performance of the process was assessed at a laboratory scale. The total energy consumption was 0.329 ± 0.08 kWh per extraction batch, corresponding to a carbon footprint of 0.12 kg CO_2_ per batch, based on Argentina’s electricity emission factor (0.387 kg CO_2_/kWh). When normalized to extraction yield, the process exhibited a relative carbon footprint of 0.0052 kg CO_2_/mg GAE, indicating favorable energy efficiency per unit of antioxidant recovered. These results demonstrate that UAE is a rapid and energy-efficient technique for the recovery of phenolic compounds from cannabis leaves, supporting their valorization within a circular economy framework. However, further studies at a pilot scale and full life cycle assessment are required to confirm the environmental performance of the process at industrial level.

## 1. Introduction

Specialized metabolites such as phenolic compounds, terpenoids, terpen-phenolics, alkaloids and pigments, which are found in medicinal plants, are responsible for their biological and pharmacological properties [1,2]. Bioactive substances isolated from plant material can exhibit antioxidant, antimutagenic, anticancer, antiemetic, antifungal and antibacterial properties, among others [3,4,5,6,7]. These substances have a variety of applications in food, cosmetics, pharmaceuticals and agro-industries, to name a few. The extraction of these compounds depends on several factors. Correctly preparing the material for extraction is just as important as selecting the extraction method [8,9]. Current preparation of the material for extraction includes drying and grinding it to a powder to facilitate the release of bioactive compounds [8]. Various conventional and unconventional extraction methods have been employed to date, achieving different levels of efficiency. Examples include ultrasound-assisted extraction, microwave-assisted extraction, and extraction using supercritical fluids [10,11,12]. Recent advances in extraction methodologies aim to enhance the extraction efficiency while reducing extraction time and solvent quantities. Other important factors in bioactive extraction include solvent type, the ratio of plant material to solvent, and temperature, among others [9,11]. Therefore, further research is required to optimize the factors related to the bioactive extraction process.

*C. sativa* L. is a plant that has a history of use in traditional medicine as well as in substance abuse. *C. sativa* is rich in chemical compounds, including polyphenols, terpenoids and terpeno-phenolic compounds such as cannabinoids, which exhibit a wide range of bioactivities [13,14,15,16,17,18,19,20]. On the other hand, the increasing awareness of the potential pharmacological benefits of *C. sativa* has led to a growing global market of cannabis-derived medicinal products. In Argentina, the category of “plant products based on cannabis and its derivatives for use in human medicine” has been established under laws 27350 and 27669, as well as Resolution 781/2022, which was subsequently amended by Provision 767/2023 of the Ministry of Health of the Nation. In its guide for the health authorization of plant-based cannabis products, the National Administration of Medicines, Foods and Medical Technology (ANMAT) defines the “plant drug” as the dried, whole or fragmented female inflorescences and accompanying upper leaves obtained from the female plant (or from plants with feminized seeds) of *C. sativa*, including all its subspecies, varieties and chemotypes [21]. At the national level, recent reports estimate that the cannabis market in Argentina could reach approximately USD 1.7 billion under a fully developed regulatory framework, with a strong contribution from the medicinal segment [22].

Thus, several cannabis strains or varieties were stabilized and propagated in different provinces of Argentina. According to ANMAT guidelines, the inflorescences, along with their upper leaves, are considered a “plant drug” and can be used in the Argentine pharmaceutical industry. In contrast, the mature leaves can be considered a byproduct of the medicinal cannabis industry and are generally discarded. The Argentine pharmaceutical industry incorporates inflorescences and upper leaves into tinctures (alcohol macerations) to obtain the resin, which is then added to oils. Some authors have reported the cannabinoid, terpene and polyphenol levels in inflorescences from Argentina varieties and some biological activities [23,24,25,26,27]. Mature leaves obtained through defoliation, pruning, or cuttings are only used for composting along with the cannabis stems and are considered waste. Some authors have demonstrated through image analysis that cannabinoids accumulate in the trichomes of the inflorescences and leaves, while phenolic compounds accumulate mainly outside the trichomes and primarily in the leaves [28]. The high proportion of leaf biomass relative to the inflorescences means that discarded leaves from Argentinian cannabis strains could be a valuable source of phytochemicals, such as phenolic compounds, for use in cosmetic products or medicinal products. It is widely known that environmental conditions generate chemical and morphological variability in plants. Furthermore, factors such as the extraction solvent, temperature, plant material/solvent (PM/S) ratio, and the extraction method (maceration, microwave, ultrasound, supercritical fluid) define the type and concentration of metabolites extracted (phytocannabinoids or phenolic compounds) and, consequently, the medicinal properties of the extracts [29]. Therefore, it is important to obtain optimized products to achieve high-quality products that offer safety and efficacy.

The aim of this study is to optimize the extraction of phenolic antioxidant compounds from the powdered leaves of *C. sativa* (INBIOFIV 00500 Tuc chemotype) grown from Tucumán, Argentina, using green solvents and ultrasound-assisted extraction (UAE), a method that is increasingly recognized for its efficiency and sustainability [29,30].

## 2. Results and Discussion

Extracts of powdered and dried leaves of cannabis grown in Tucumán, Argentina, with particle size distribution between 74 and 840 µm (Table 1) were prepared using various powder-to-solvent ratios and different solvents, via conventional (maceration) and unconventional methods, such as ultrasound-assisted extraction (UAE). This technology is based on the principle of acoustic cavitation, which can damage the cell walls of the plant matrix and favor the release of bioactive compounds. The UAE is considered a sustainable alternative that requires a moderate investment of solvent and energy. The extracts obtained by UAE were characterized chemically, and their antioxidant properties and toxicity were determined.

The efficiency of the UAE using cannabis powdered leaves was evaluated under the influence of three variables: extraction time (1, 6 or 12 min); solid/liquid ratio (1.25, 2.50 or 5 g of plant material per 50 mL of solvent); and solvent type (distilled water or ethanol at various concentrations (40, 60 or 96%). The phenolic and flavonoid content of each extract was determined. Response surface methodology (RSM) was then applied to identify the optimal extraction conditions of polyphenolic compounds.

### 2.1. Total Phenolic Compound (TPC) and Flavonoid (TF) Content in Extracts Obtained by UAE at Different Conditions

**Figure 1:**
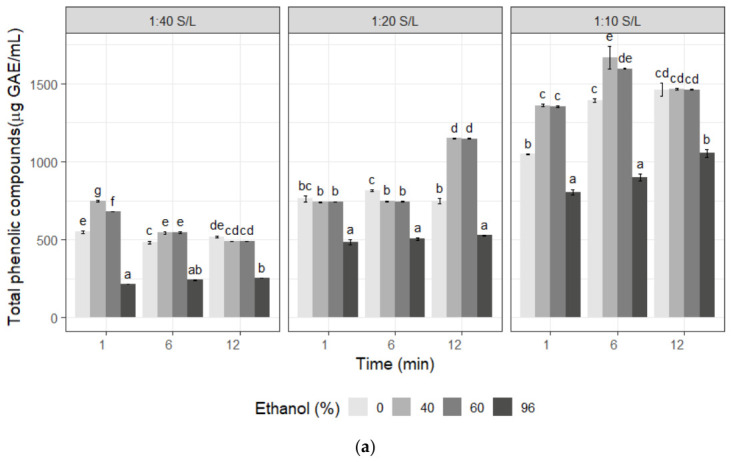

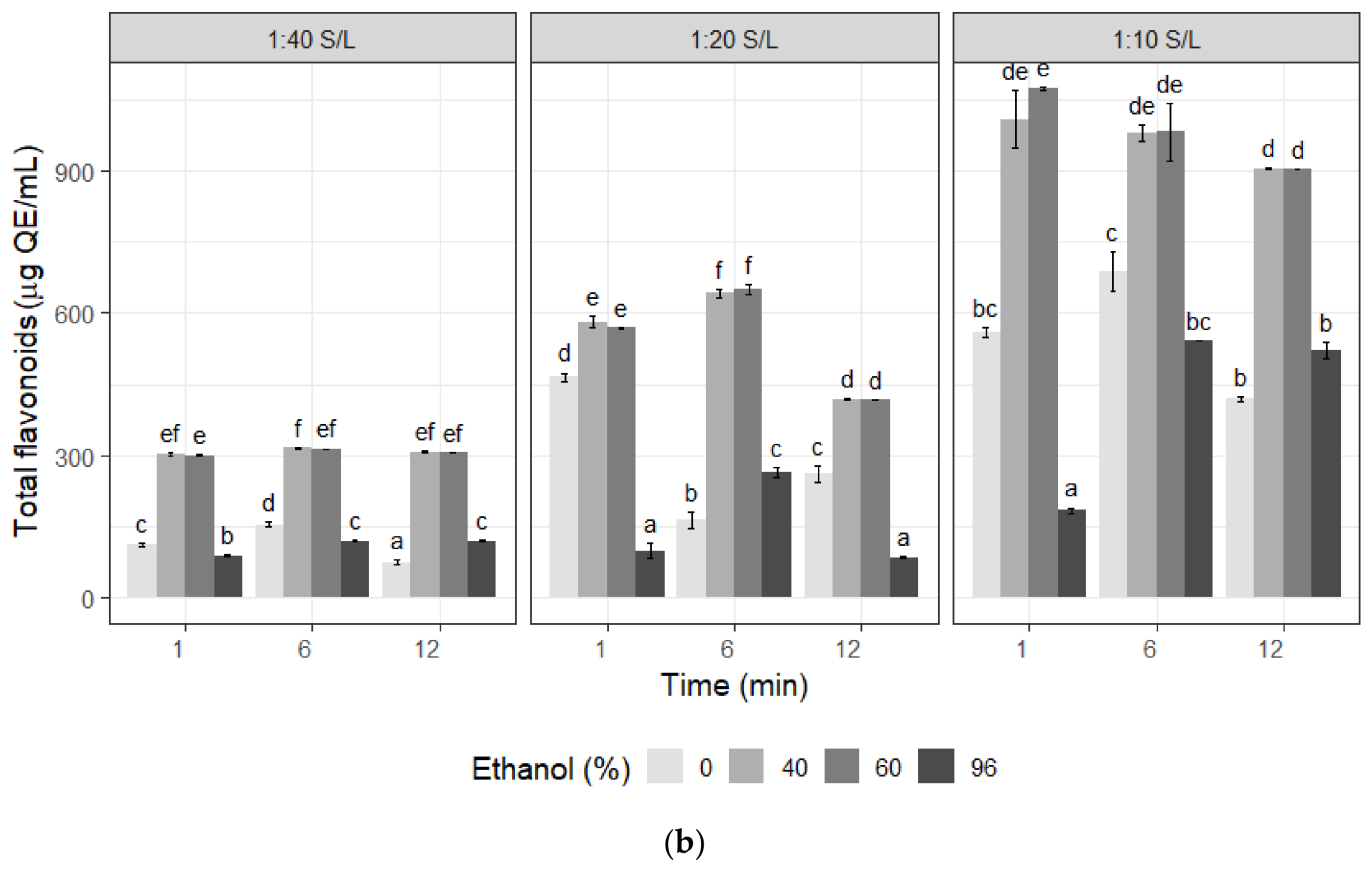
Phytochemical screening of *Cannabis sativa* extracts. (**a**) Total phenolic compounds (µg GAE/mL); (**b**) total flavonoid content (µg QE/mL). GAE: Gallic acid equivalent; QE: quercetin equivalent; S/L: solid-to-liquid ratio. Different letters above the bars represent statistically significant differences at *p* ≤ 0.05, according to Tukey’s test.

The Argentine cannabis leaf extracts exhibited variable total phenolic content (TPC) levels ranging from 214.4 µg GAE/mL to 1598.2 µg GAE/mL of extract (25–135 mg GAE/g DW) as well as total flavonoid (TF) levels ranging from 75.7 to 1110 µg QE/mL (5.45–79.28 mg QE/g DW), varying according to the experimental conditions. These results are shown in Figure 1.

A solid-to-solvent ratio of 1:10 proved to be the most efficient for extracting TPC and TF by UAE. The extracts corresponding to this ratio exhibited the highest TPC and TF concentrations, compared to those obtained using low S/L ratios (1:20 and 1:40). Ultrasound-assisted extraction efficiency is strongly dependent on cavitation phenomena, which enhances mass transfer and promote cell wall disruption, facilitating the release of intracellular compounds such as phenolics. At a 1:40 ratio, the cavitation intensity per unit volume likely decreases. Because the solid is more dispersed, there are fewer collisions and less localized cavitation-induced turbulence, resulting in lower mass transfer efficiency near the surface of the solid. In summary, the system is less efficient both energetically and mechanically than at a 1:10 ratio [29,30,31].

Notably, the highest concentrations of TPC and TF were obtained using 40% and 60% ethanol with an extraction time between one and six minutes, reaching approximately 1600 µg GAE/mL for TPC and over 1000 µg QE/mL for TF. During the first six minutes, cavitation effects promoted cell disruption and the rapid release of readily accessible cannabis phenolics (washing stage). After this period, the polyphenol release process slowed down and was evidently controlled mainly by diffusion. Consequently, extending the extraction time to 12 min does not significantly increase the yield, since most of the extractable phenolic compounds have already been recovered and the system approaches solid–liquid equilibrium. This aligns with previous reports, stating that shorter extraction times help to preserve the quality of bioactive compounds, whereas prolonged times may promote degradation and reduce bioactivity potency. Furthermore, the choice of solvent and extraction conditions significantly affects the efficiency with which bioactive compounds are recovered [29,30,31,32,33,34].

In terms of solvent composition, a mixture of 40% and 60% ethanol produced higher yields of TPC and TF than water or 96% ethanol at all evaluated solid-to-liquid (S/L) ratios and extraction times. This confirms that this mixture is more effective at extracting phenolic and flavonoid compounds of different polarities. Other authors reported that in the cannabis leaf extracts obtained with 50% ethanol, the highest flavonoid content was 11.21 mg QE∙g^−1^ DW for young plants and 5.21 mg QE∙g^−1^ DW for mature plants [32]. The levels of flavonoids in mature leaves of Argentine strains were around 15 times higher than those reported by Kobus et al. [32]. Studies conducted by Izzo et al. [33] showed that the average flavonoid content in the inflorescences of cannabis was about 0.62 mg/g DW. The different phytochemical compositions could be explained by the cannabis cultivars and the growing conditions used in each case [35].

The analysis indicates that the 1:10 S/L ratio was the most efficient, and that the highest concentrations of TPC were obtained using 40 and 60% ethanol. This suggests that this combination optimizes both ultrasonic cavitation and the solubility of the metabolites of interest.

**Figure 2:**
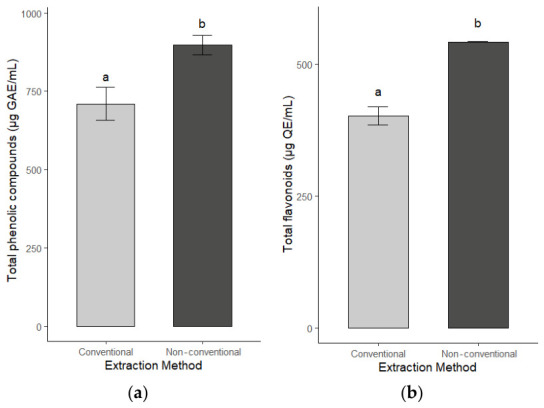
Total phenolic compound (**a**) and flavonoid (**b**) content of *Cannabis sativa* extracts obtained by conventional (maceration) and non-conventional (ultrasound) extraction. Results are expressed as mean ± standard deviation (*n* = 3). Different letters indicate significant differences between treatments (*p* ≤ 0.05).

It was decided to compare the results of UAE (ratio 1:10, six minutes and 96% ethanol) with those obtained using a conventional method by maceration (ratio 1:10 with 72 h of incubation and 96% ethanol). This methodology is described in the Argentine Pharmacopoeia to produce phytotherapeutic tinctures [36]. The results indicated that the UAE was more efficient at extracting total phenolic compounds and flavonoids from *C. sativa* leaves (TPC (ultrasound) 900 µg GAE/mL and TF (ultrasound) 540 µg QE/mL and TPC (maceration) 740 µg GAE/mL and TF (maceration) 410 µg QE/mL) (Figure 2).

These results confirm that ultrasound applications improve metabolite release, constituting an effective and rapid alternative for phytochemical extraction.

### 2.2. Analysis by TLC and HPLC-DAD

**Figure 3:**
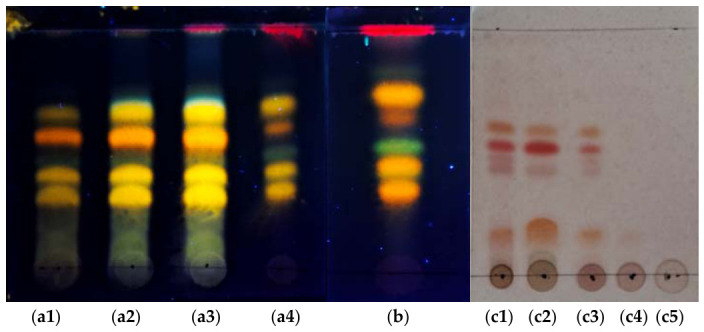
Phytochemical profile of *Cannabis sativa* extracts analyzed by TLC. (**a1**–**a4**,**b**) Extracts developed with Neu (NP/PEG) reagent and visualized under UV light at 365 nm: (**a1**) UAE, distilled water; (**a2**) UAE, 40% ethanol; (**a3**) UAE, 60% ethanol; (**a4**) UAE, 96% ethanol. (**b**) Conventional maceration, 96% ethanol. (**c1**–**c5**) Extracts revealed with Fast Blue BB: (**c1**) Conventional maceration, 96% ethanol; (**c2**) UAE, 96% ethanol; (**c3**) UAE, 60% ethanol; (**c4**) UAE, 40% ethanol; (**c5**) UAE, distilled water.

The profiles of the extracts were evaluated by TLC. The presence of yellow, orange, green, and light-blue bands indicate the presence of flavonoids and phenolic acids (Figure 3). This pattern suggests that the analyzed extracts contain a complex mixture of phenolic compounds. Using ultrasound-assisted extraction with water and ethanol in different gradations enables a greater variety of compounds with different polarities to be recovered, resulting in a more intense and diverse chromatographic profile. The cannabinoid compound profiles showed that extracts obtained using higher proportions of ethanol, particularly ethanol 96%, produced intense reddish-orange bands, which are indicative of a higher cannabinoid concentration. In contrast, more aqueous extracts produced noticeably fainter bands, highlighting the lower extraction efficiency of lipophilic cannabinoids in these media. As with flavonoids and phenolic acids, this chromatographic pattern showed that extraction of cannabinoids is highly dependent on the polarity of the solvent (Figure 3).

**Figure 4:**
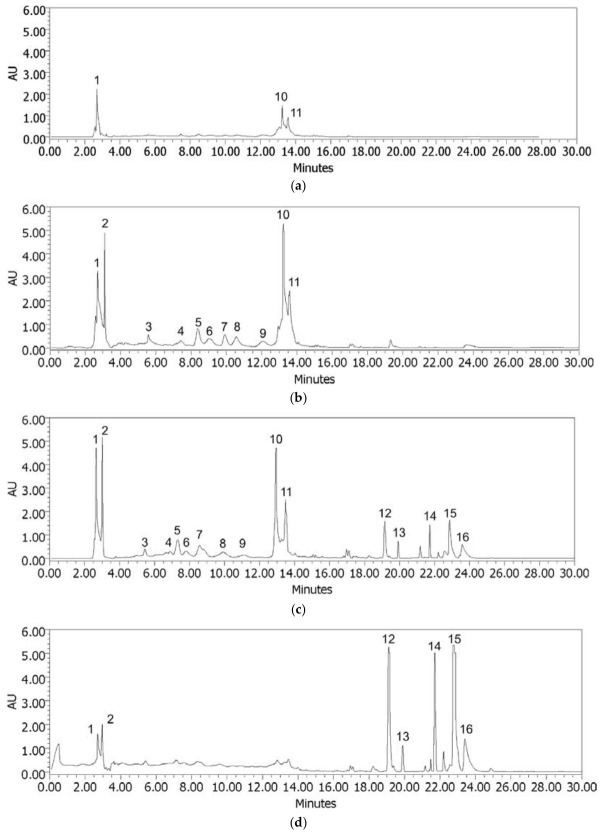
HPLC-DAD profiles of ultrasound-assisted extraction (UAE) in 1:10 S/L ratio, using different solvents: (**a**) Distilled water, (**b**) ethanol 40%, (**c**) ethanol 60% and (**d**) ethanol 96%. Fingerprints were registered at MaxPlot.

Figure 4 and Table 2 show the HPLC-DAD profiles obtained from cannabis extracts of cultivars from Tucumán (Argentina) using different solvents and a 1:10 S/L ratio and UAE. Ultrasound-assisted 40% ethanol extraction produced eleven peaks in the first 14 min of elution, while water alone can extract only highly polar compounds with UV profiles corresponding to phenolic compounds [37]. In the ethanolic extract (40%), peaks with retention times between 7.4 and 13.5 min were identified as kaempferol, diosmetin, vitexin, isovitexin, rutin, luteolin and apigenin. Some of these flavonoids have also been reported in methanol extracts of cannabis leaves from various regions worldwide [13]. Kaempferol, rutin, luteolin, apigenin, and apigenin C-glycosides possess important bioactive properties, such as antioxidant and anti-inflammatory effects [38,39,40]. Although phenolic acids and cannabinoid acids were found in aqueous extracts of cannabis varieties grown in other parts of the world [41], these types of compounds were not identified in aqueous extracts of leaves from the local Argentine variety from Tucuman (Table 2).

Interestingly, the extract obtained using 60% ethanol showed a greater number of peaks (peaks 12–16) identified as CBDA, CBD, THC and THCA (Table 2). In extracts obtained with ethanol 96% as solvent, only cannabinoids such as CBDA, CBD, THC and THCA were identified (Figure 4). This highlights the importance of biomass concentration, in addition to the type of solvent, for the recovery of these non-polar compounds. Similar results were obtained for other chemotypes using methanol and ultrasound [42]. It is important to note that extracts obtained with a 1:10 ratio in a lower alcohol concentration (40%) yield extracts enriched in phenolic compounds, primarily flavonoids, and free of THC-type cannabinoids, which are considered psychoactive. According to Argentine regulations, these extracts could be used in the development of THC-free cannabis-based medicinal products [22].

### 2.3. Optimization of Ultrasound-Assisted Extraction

Based on the experimental results described in Section 2.1. and previous reports, the independent variables and operational ranges for the implementation of the response surface methodology (RSM) were selected. An unbalanced full factorial design was selected and adjusted by Type III ANOVA to maintain statistical validity of the model. Thirty-one experimental groups, including five center points to estimate pure error, were obtained. The total phenolic and flavonoid content of each extract was determined. The results are shown in Table 3.

A reduced quadratic mathematical model was used for adjustment, and the coefficients of the second-order polynomial model were calculated (Table 4). These coefficients were then used to predict the content of phenolics and flavonoids. The analysis of variance (ANOVA) showed F-values of 267.23 and 49.24 for TPC and TF, respectively, indicating that the models were statistically significant. The R^2^ values (0.99 and 0.89 for TPC and TF, respectively), adjusted R^2^ (0.98 and 0.87 for TPC and TF, respectively), and predicted R^2^ (0.98 and 0.84 for TPC and TF, respectively) suggested that the statistical models had been correctly chosen for both variables.

The multiple-regression equations for the extraction content of total phenolic compounds (Y_TPC_) and flavonoids (Y_TF_) are shown below, with A (S/L ratio), B (extraction time), and C (solvent):

Sqr (Y_TPC_) = 27.28 − 7.74 A − 0.0808 B − 3.12 C + 5.10 A^2^ + 0.08615 B^2^ − 5.98 C^2^

Y_TF_ = 559.14 − 257.35 A − 37.18 C + 185.03 A^2^ − 417.78 C^2^

**Figure 5:**
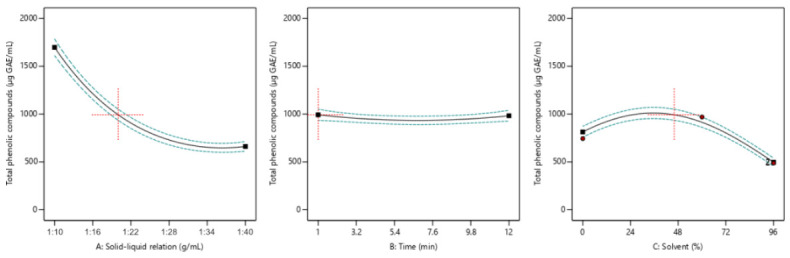
Predicted response profiles for total phenolic compounds (TPC) showing the effect of solid-to-liquid ratio (**A**), extraction time (**B**), and ethanol concentration (**C**). The black line represents the predicted response, and the blue lines indicate the 95% confidence bands.

The terms A and A^2^ had a significant effect (*p* ≤ 0.05) on both modeled variables, demonstrating the importance of the S/L ratio in this extraction process. In contrast, although extraction time (B) did not have a statistically significant linear effect, it was included in the hierarchical TPC model because the values of its quadratic coefficient (B^2^) showed a *p*-value ≤ 0.05, indicating a curvature effect. Figure 5 shows that variations in extraction time within the studied range (1–12 min) did not affect the extraction yield, whereas the solid-to-liquid ratio (A) and ethanol concentration (C) had a significant effect on the response variable. For this, during optimization, the time factor was held constant at a minimum level of one minute, while the combined effects of the other two factors on the response variable were analyzed. Previous studies have confirmed that prolonged exposure to ultrasound decreases extraction efficiency and can result in damage to plant tissue as well as the degradation of thermolabile compounds [30,32,41]. Based on this, minimizing exposure time to ultrasonic waves was used as a criterion. Furthermore, coefficients C and C^2^ were statistically significant for phenolic compounds. However, in the flavonoid prediction model, only C^2^ had a *p*-value ≤ 0.05, so its linear coefficient had to be included to maintain the model hierarchy. No statistically significant interactions were found between these factors.

**Figure 6:**
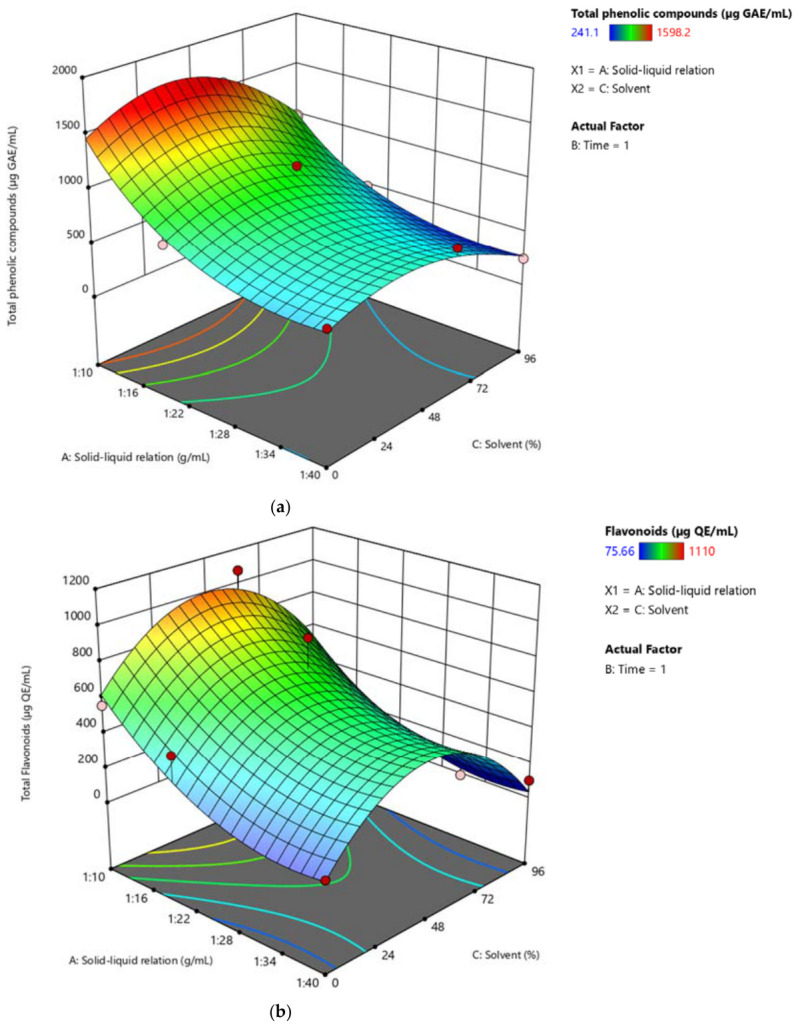
Response surface plots of ultrasound-assisted extraction (UAE). The effect of the S/L ratio and ethanol concentration on the extraction of (**a**) total phenolic compounds and (**b**) flavonoids. The blue and red areas represent low and high yields, respectively, of total phenolic compounds and flavonoids. GAE: Gallic acid equivalents; QE: quercetin equivalents.

The 3D surfaces generated (Figure 6) clearly visualized the optimal regions for each response variable, corresponding to the red areas in Figure 6a,b. TPC showed an optimum at decreasing S/L ratios and intermediate ethanol content. Similarly, TF content was favored under the same conditions.

Considering the linear regression equation for TF, the linear and quadratic terms of ethanol concentration showed negative correlations, suggesting that increasing these variables can promote flavonoid extraction to a certain extent. This effect is particularly evident in Figure 6b, where the TF yield increases within the 0–40% range and then decreases with further increases in ethanol concentration.

This could indicate that the flavonoids are predominantly polar, as observed in Figure 5 and Table 2.

Both 3D plots show that the regions of optimal performance for TPC and TF occur at higher ratios of plant material/solvent and decrease significantly as the ratio of grams of plant material per milliliter of solvent diminishes.

#### Model Validation

The optimal conditions to maximize TPC and TF extraction were obtained with an extraction time of 1 min, a ratio between cannabis powder and solvent of 1:10, and 46% ethanol concentration. Under these conditions, a Derringer global desirability value of 0.964 was achieved, indicating that the model can predict the evaluated responses simultaneously. The validity of the RSM-derived model was evaluated by performing experimental validation for both variables, in triplicate, under the optimal conditions suggested by the software. The responses obtained were then compared with the model’s predicted values, considering a two-tailed prediction interval of 95%.

As shown in Table 5, the predicted value for total phenolic compounds was 1697.9 µg GAE/mL, while the experimental value was 1746.83 µg GAE/mL. This result lies within the prediction interval (1574.91–1823.81 µg GAE/mL), demonstrating the model’s ability to accurately predict this variable.

For flavonoids, the predicted value was 1002.35 µg QE/mL. The average of the experimental triplicate was 858.41 µg QE/mL, which falls within the predicted interval of 841.77–1162.92 µg QE/mL. This validates the robustness of the proposed statistical model. A similar concentration of flavonoids was described by other authors in another cannabis variety [29].

### 2.4. Antioxidant Activity

**Figure 7:**
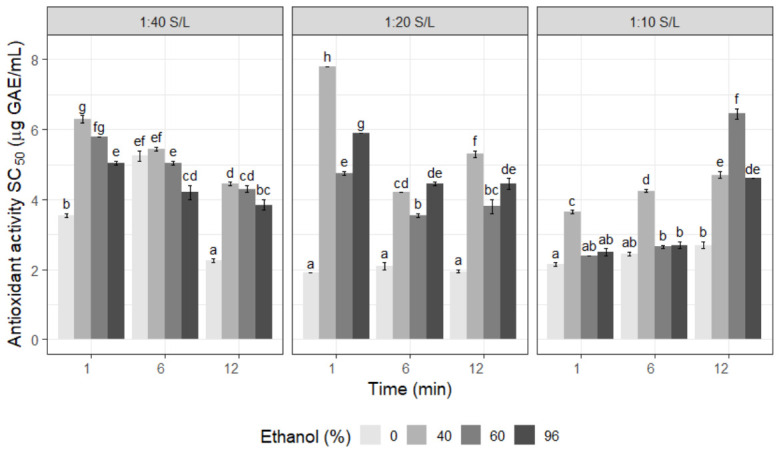
Antioxidant activity of *Cannabis sativa* extracts by ABTS^•+^ assay. The graph shows the SC_50_ values (µg GAE/mL). GAE: Gallic acid equivalents; S/L: solid-to-liquid ratio. Different letters above the bars represent statistically significant differences at *p* ≤ 0.05, according to Tukey’s test.

Reactive oxygen species (ROS) are a group of highly reactive oxygen radicals and molecules, characterized by their strong oxidizing properties. They produce oxidative stress, which has harmful effects on biomolecules such as lipids, proteins and DNA and affects several physiological functions. Oxidative stress is a common feature of several chronic diseases, including cardiovascular disease, cancer, diabetes, and obesity, and antioxidant intake in the form of vitamins, selenium, and carotenoids can reduce redox imbalance. Several authors have reported that CBD readily targets oxidative signaling and ROS production [43]. However, according to the literature, phenolic compounds may be responsible for over 90% of the antioxidants ingested daily [44]. The concentration of phenolic antioxidants from different commodities and by-products has been widely studied and compiled in various databases and reviews [44,45,46,47]. The antioxidant potential of all cannabis extracts was determined by the scavenging concentration of 50% (SC_50_) ABTS cation radical (ABTS^•+^). The SC_50_ ABTS values ranged from 1.5 to 5.4 μg GAE/mL in the different extracts, indicating high antioxidant potential (Figure 7). Its antioxidant potency was compared with that of some of the flavonoids identified in cannabis extracts. The SC_50_ values were like those of luteolin and kaempferol (2.7 ± 0.4 µg/mL and 4.9 µg GAE/mL, respectively). The antioxidant properties of all flavonoids, which are present in the analyzed extracts, i.e., kaempferol, diosmetin, vitexin, isovitexin, rutin, luteolin and apigenin, have also been previously reported and their action mechanisms determined [48,49,50,51].

Extractions in ethanol 60 and 96% showed high antioxidant potency, probably due to the content of cannabinoids demonstrated (Figure 2).

The antioxidant activity of the optimized extract was subsequently measured, yielding an average SC_50_ value of 2.9 ± 0.6 µg GAE/mL comparable to luteolin. Thanarukwuttikorn et al. [33] and Matešić et al. [52] reported a positive correlation between ABTS^•+^ scavenging activity and total flavonoid content.

To determine if the cannabis leaf extracts, in addition to transferring electrons and producing the reduction of oxidizing molecules, can inhibit enzymes that are linked to the generation of ROS, the effect of the standardized extract on xanthine oxidase (XO) was determined. XO is an enzyme involved in the metabolism of purines that catalyzes the oxidation of xanthine to uric acid, generating superoxide anions as a byproduct. This has been associated with acute and chronic inflammatory conditions and aging-related processes [6]. In this regard, the inhibition of xanthine oxidase represents an additional mechanism to reduce ROS production and mitigate oxidative damage. The optimized extract also demonstrated the ability to inhibit xanthine oxidase activity, showing 61.9 ± 9.6% inhibition at 8 µg GAE/mL, which indicates a strong inhibitory effect at low concentration. In comparison, allopurinol (XO inhibitor) exhibited an IC_50_ value of 50 µg/mL. This reinforces its antioxidant potential through both direct (free-radical scavenging) and indirect (enzyme inhibition) mechanisms. Although the cannabis leaves are considered industrial waste, this biomass could be used to obtain extracts enriched with bioactive compounds capable of helping to reduce oxidative stress and inflammatory processes. Our results could encourage the cultivation of this variety in Argentina, particularly in the northwestern region. This could ensure a steady supply of antioxidant-rich cannabis leaves for use in cosmetic or medicinal products, promoting sustainability by minimizing waste. It is worth noting that this is the first report on the antioxidant capacity of extracts obtained from leaves from Argentine cannabis varieties by UAE. The antioxidant capacity of inflorescence extracts from other varieties grown in Tucumán has been previously published [22].

### 2.5. Acute Toxicity

*Artemia salina* is a model alternative to the use of experimental animals to evaluate. acute toxicity. The present study was conducted to determine the toxicity of the optimized cannabis extract in a range of concentrations tenfold higher than the concentration necessary to show antioxidant capacity (SC_50_ values). Results from the *A. salina* assay showed that Argentine cannabis extract was not toxic in the range of concentrations analyzed (5–100 μg GAE/mL equivalent to 50–1000 mg DW), exhibiting mortality of less than 10% at all concentrations tested. Similar results were reported by Di Simone et al. [40] for hemp-derived extracts. Meyer’s and Clarkson’s classification categorizes substances as non-toxic for LC_50_ values above 1000 μg/mL, low-toxic for LC_50_ values between 500 and 1000 μg/mL, medium-toxic for LC_50_ values between 100 and 500 μg/mL, and highly toxic for LC_50_ values between 0 and 100 μg/mL [53,54]. Therefore, considering the LC_50_ value is higher 1000 μg/mL, the optimized extract from cannabis waste can be considered non-toxic in the Artemia toxicological model. Other toxicity models could be used in the future.

### 2.6. Relative Energy Efficiency and Carbon Footprint to Optimized Extract

This study analyzed the environmental performances of laboratory-scale processes for the extraction of polyphenols from cannabis leaf powder. The system included drying, grinding and ultrasound-assisted extraction. According to the literature, electricity consumption and solvent use represent the main environmental hotspots in extraction processes [55,56]. The total energy consumption of the process was 0.329 ± 0.08 kWh per extraction batch. Considering an emission factor of 0.387 kg CO_2_/kWh for Argentina’s electricity matrix, the total carbon footprint (TCF) associated with the process was estimated at 0.127 kg CO_2_ per batch for UAE and 0.2664 kg CO_2_ for extraction by maceration. To enable comparison, the results were normalized to the total phenolic content (TPC), yielding a relative carbon footprint (RCF) of 0.0028 kg CO_2_/mg GAE, while the extraction by maceration yielded 0.0072 kg CO_2_/mg GAE. These results demonstrate the environmental performance of the process in terms of both energy efficiency and climate impact per unit of antioxidant compounds, particularly considering that the raw material corresponds to a byproduct of the medicinal cannabis industry. While synthetic antioxidants such as Butylated hydroxytoluene are associated with fossil-based feedstocks and energy-intensive chemical synthesis, bio-based antioxidants such as phenolic compounds derived from *Cannabis sativa* are not inherently carbon-neutral. Their environmental impact strongly depends on cultivation conditions, which may dominate life cycle greenhouse gas emissions, particularly in energy-intensive production systems.

## 3. Materials and Methods

### 3.1. Chemicals

Folin–Ciocalteu reagent, Fast Blue BB, aminoethyl ester, diphenylboric acid, AlCl_3_ and reference compounds (luteolin, vitexin, isovitexin, apigenin, kaempferol, gallic acid, and quercetin) were acquired at Sigma Aldrich, St. Louis, MO, USA. Methanol and acetic acid (Biopack, Buenos Aires, Argentina). THC (tetrahydrocannabinol), CBG (cannabigerol), CBD (cannabidiol), CBN (cannabinol), CBC (Cannabichromene), CBDA (Cannabidiolic acid), ∆9-THCA (∆9-Tetrahydrocannabinolic acid), CBGA (Cannabigerolic acid), and CBCA (Cannabichromenic acid) were acquired at Restek-Jenck, Buenos Aires, Argentina. The flavonoid standard Cannflavin A was purchased from Cayman Chemical, Ann Arbor, MI, USA.

### 3.2. Plant Material

The plant material (leaves) was obtained from *Cannabis sativa* cultivated at the INBIOFIV in San Miguel de Tucumán, Tucumán, Argentina (Res.2021 9APN SAS#MS). The voucher number was assigned and is recorded in the INBIOFIV herbarium database: INBIOFIV 00500. The plant material was dried in a forced-air oven at 40 °C under controlled-humidity conditions until a constant weight was achieved. Once dried, the plant material was ground using a Numak F100 grinder (Numak, London, UK). The powder was stored in vacuum-sealed bags at approximately 25 °C until extraction.

#### Particle Size Analysis

Three grams of leaf powder were weighed and placed in a Zonytest LR 2006 digital sieve shaker (Zonytest, Buenos Aires, Argentina), equipped with a set of five mesh sieves with openings measuring 840 µm, 500 µm, 149 µm, 105 µm, and 74 µm to analyze the particle size distribution of the plant powder. The sample was shaken for five minutes. The dust fractions retained on each sieve were weighed, and the corresponding retention percentage was calculated. This process was repeated three times for each type of powder analyzed.

### 3.3. Extraction of Bioactive Compounds

#### 3.3.1. Ultrasound Probe-Assisted Extraction (UAE)

Extraction mixtures containing 1.25, 2.50, or 5.0 g of ground leaves and 50 mL of solvent (water or 40%, 60% and 96% ethanol) in a 100 mL beaker were prepared. The mixtures were then subjected to ultrasound-assisted extraction using a 0.7 mm diameter, 95 mm length titanium ultrasonic probe (UP200St, Hielscher Ultrasonics GmbH., Teltow, Germany). The extraction conditions were selected based on preliminary experimental trials and reports on conditions for obtaining polyphenols by ultrasound to minimize the degradation or oxidation of compounds due to the generation of hydroxyl radicals [28]. The probe was immersed directly in the beaker to ensure direct contact with the sample. The beaker was then placed in a water bath to stabilize the temperature at 30 °C. The samples were sonicated using the following processor arrangement: 5 s on and 10 s off. Effective operation times were 1, 6, and 12 min at an amplitude of 70% (corresponding to the ultrasound intensity) and maximum power of 100 W operating at a frequency of 26 kHz. The mixtures were then filtered under vacuum using Whatman N° 1 filter paper and stored in a freezer at −20 °C away from light until further analysis. The extractions were carried out in triplicate.

The optimal extraction conditions were determined by response surface methodology (RSM) based on the experimental design, which involved a 1:10 solid-to-liquid ratio and 46% ethanol as the combination that maximizes phenolic and flavonoid recovery. The conditions were subsequently applied to obtain the optimized extract using the same operating parameters described above.

##### Experimental Design and Statistical Analysis

The experiment was conducted based on an experimental design created using Design-Expert v13 software (Stat-Ease, Minneapolis, MN, USA). The levels of the independent variables were predefined based on previous experimental conditions to obtain cannabinoid free extracts. The selection of solvent composition (ethanol–water mixtures) was based on previous reports describing the efficient extraction of phenolic compounds from *Cannabis sativa* and other plant matrices using hydroalcoholic systems, as well as on the need to employ food-grade, environmentally friendly solvents. The chosen range was intended to balance polarity to maximize the recovery of antioxidant compounds. Regarding ultrasound-assisted extraction parameters, although not all variables were systematically optimized, the selected conditions were defined based on a combination of literature data and experiments carried out in our laboratory. These preliminary trials allowed us to identify operational ranges that ensure detectable extraction yields while avoiding compound degradation. For this, an unbalanced full factorial design was selected and adjusted by Type III ANOVA to maintain statistical validity of the model. The following independent variables were included in the design: S/L ratio, extraction time, and ethanol concentration. The levels for each factor are shown in Table 6. Thirty-one experimental groups were obtained through the response surface experimental design, including five center points to estimate pure error. The dependent variables selected for the experimental design were the yield of total phenolic compounds (μg GAE/mL) and total flavonoids (μg QE/mL).

A second-order polynomial model was used to explain the effect of the independent variables on each response, according to the following general equation:

*y* = *β*_0_ + *β*_1 A_ + *β*_2 B_ + *β*_3 C_ + *β*_1-2 AB_ + *β*_1-3 AC_ + *β*_2-3 BC_ + *β*_1-2-3 ABC_ + *β*_1-1 A_^2^ + *β*_2-2 B_^2^ + *β*_3-3 C_^2^ + *e*

Here, *y* represents the response variables (total phenolic or total flavonoid content); **β_0_** represents the constant; **A**, **B**, and **C** are the independent variables; and *β* _1, 2, 3_, *β* _1-2, 1-2, 2-3_, *β* _1-2-3_, and *β* _1-1, 2-2, 3-3_ are the linear, double-interactive, triple-interactive, and quadratic coefficients, respectively.

The experimental data were evaluated using analysis of variance (ANOVA). The statistical significance of the regression coefficients was verified using an F-test, with *p*-values below 0.05 being considered significant.

The model corresponding to the response variable TPC showed heteroskedasticity in the residuals, so a Box–Cox transformation, as suggested by the software, was performed. The optimal lambda value suggested was λ = 0.5, corresponding to a square-root transformation, applied as √Y (k = 0). This transformation stabilized the variance and improved the model fit.

The Derringer desirability prediction tool was used to estimate optimal extraction conditions, seeking the maximum achievable response for each independent factor. The validity of the developed model was then assessed by comparing the experimental values with the predicted values.

#### 3.3.2. Maceration

For the conventional extraction process, 10 g of plant material was weighed and macerated with 100 mL of 96% ethanol. The resulting mixture was placed in a shaking heating bath (Lab Companion AAH44113U BS 11, Daejeon, South Korea) and extracted for 72 h at 30 °C and at 105 rpm, in accordance with the tincture preparation procedures set out in the Argentine Pharmacopoeia [36]. The use of 96% ethanol was selected to ensure comparable conditions and is consistent with the Argentine Pharmacopoeia for the preparation of the phytotherapeutic tinctures. This comparison was conducted as a preliminary assessment of extraction efficiency between conventional maceration and ultrasound-assisted extraction, rather than to evaluate solvent effects.

After extraction, the mixture was then filtered under vacuum conditions using Whatman N° 1 filter paper, and the extract was stored in a freezer at −20 °C away from light until further analysis. Extractions were carried out in triplicate.

### 3.4. Phytochemical Analysis

#### 3.4.1. Extraction Yield

To determine the extraction yield in dry weight (DW), the solvent was completely evaporated using a rotary evaporator (BÜCHI R-110, Flawil, Switzerland). The remaining fraction was then frozen at −20 °C and freeze-dried (L-M10-A-E50-CRT, RIFICOR, Buenos Aires, Argentina) to remove the water content and produce a solid residue. The dry weight (DW) was determined using the gravimetric method. The results were expressed as milligrams of DW per milliliter of the extract.

#### 3.4.2. Total Phenolic Compounds (TPC) and Total Flavonoid (TF) Content

The total phenolic compound content was determined using the Folin–Ciocalteau reagent (Folin–Ciocalteau reagent, Merck, Darmstadt, Germany) according to Singleton et al., 1999 [57]. Total flavonoids were estimated using diphenylboric acid 2 aminoethyl ester solution reagent (1% *v*/*v*) according to Teh and Birch, 2014 [58]. Absorbance was recorded in a UV/visible spectrophotometer (Jasco V-630, Thermo Fisher Scientific, Tokyo, Japan). Solvent controls (water and ethanol at 40%, 46%, 60% and 96% concentrations) were performed to check for any possible interference. All determinations were performed in triplicate, and the results were expressed as µg equivalents of gallic acid (µg GAE/mL) or quercetin (µg QE/mL) equivalents per milliliter of extract, for polyphenols and flavonoids, respectively. The results were also expressed in g GAE/g dry weight and g QE/100 g dry weight.

#### 3.4.3. Phenolic Compound and Cannabinoid Profile

The chemical profiles of the phenolic compounds and cannabinoids in all the *C. sativa* extracts were determined using high-performance liquid chromatography (HPLC)–diode array detection (DAD). Prior to the injection of 20 μL, the extracts were filtered through a 0.45 μm nylon filter. HPLC analysis was performed using a Waters 1525 binary pump system with a Waters 1500 series column heater and a Waters 2998 photodiode array detector (PDA). Separation of the phenolic compounds and cannabinoids was performed using a YMC—C18 column (250–4.6 mm, i.d. 5 mm) at 32 °C. A binary gradient solvent system consisting of solvent A (0.1% formic acid in water) and solvent B (acetonitrile) was used. The gradient started at 12 to 18% B (0–2 min), then increased to 18% B (2–9 min), then increased further to 18 to 80% (9–13 min), then increased to 80% B (13–17 min), 80% to 100% B (17–18 min), and finally 100% B (18–24 min) at a flow rate of 1.0 mL/min. PDA acquisitions were performed from 200 to 600 nm, and the chromatogram was integrated at 290 nm. The individual compounds were identified by comparing their retention times and UV spectral data with the commercial standards of phenolic compounds (Sigma-Aldrich, St. Louis, MO, USA; and Cayman Chemical, Ann Arbor, MI, USA) and cannabinoids (Restek-Jenck, Buenos Aires, Argentina).

### 3.5. Analysis by Thin-Layer Chromatography (TLC)

#### 3.5.1. Analysis of Phenolic Compounds

An aliquot (10 μL, 1:5 dilution) of each extract was spotted onto a Silica Gel F254 plate (Merck Brand, Darmstadt, Germany). The TLC was developed using a mobile phase consisting of formic acid, acetic acid, ethyl acetate and distilled water (1.1:1.1:10:2.7; *v*/*v*/*v*/*v*). The compounds were revealed using an NP/PEG reagent containing 1% aminoethyl ester of diphenylboric acid (NP) (Sigma_Aldrich, St. Louis, MO, USA) in methanol and polyethylene glycol (PEG) [59] and visualized under ultraviolet light at 254 and 365 nm using a UV lamp (Model 5L-58-Mineralight, Upland, CA, USA).

#### 3.5.2. Analysis of Cannabinoids

An aliquot of 10 μL (1:5 dilution) of each extract was spotted on a Silica Gel F254 plate. The TLC was developed using a mobile phase consisting of hexane and ethyl ether (8:2; *v*/*v*). The cannabinoid compounds were revealed and visualized using Fast Blue BB in 0.3% methanol, followed by sodium hydroxide at a concentration of 0.1 M to intensify the visualization of the bands [59].

### 3.6. Biological Activity

#### 3.6.1. ABTS^•+^ Radical Cation-Scavenging Activity

The test was carried out using the technique described by Re et al., 1999 [60] against the ABTS^•+^ radical cation. Two hundred microliters of an ABTS^•+^ solution (absorbance of 0.7 at 750 nm) was added to different dilutions of the extracts to a final volume of 200 µL. Absorbance was measured at 734 nm six minutes after the start of the reaction using a Microplate Reader (MultiskanGo, Thermo Fisher Scientific, Waltham, MA, USA). The percentage of free-radical removal was calculated using the following formula:

% scavenging = [(A_0_ − A_s_)/A_0_] × 100

where A_0_ is the absorbance of the control without extract and with an equivalent amount of solvent, and A_s_ is the absorbance in the presence of the extract. The SC_50_ is defined as the concentration of the extracts, in micrograms per milliliter (µg/mL), required to eliminate 50% of the ABTS^•+^ free radicals. Luteolin and apigenin were used as positive control. All assays were performed in triplicate.

#### 3.6.2. Xanthine Oxidase Assay

The inhibitory effect of the extracts on xanthine oxidase activity was evaluated following the method described by Perez et al. (2018) [61]. The effects of different quantities of extracts mixed with sodium phosphate buffer (200 mM, pH 7.5) and 30 µL of xanthine oxidase (0.1 U/mL) were studied. The mixture was pre-incubated at 25 °C for 15 min. The reaction was initiated by adding 60 µL xanthine (1 mM) to a final volume of 180 µL, and further incubated for 30 min at 25 °C. This reaction was determined spectrophotometrically using Microplate Reader (MultiskanGo, Thermo Fisher Scientific, Waltham, MA, USA) by measuring the synthesis of uric acid from xanthine. The inhibition percentage was calculated using the following formula:

$$\%\;\mathrm{inhibition}\;=\;\left(\frac{C100\%-M}{C100\%}\right)\;\times \;100$$

### 3.7. Toxicity Assessment

The acute toxicity of the extracts was evaluated using the crustacean *Artemia salina* as the test organism [62]. *A. salina* cysts were incubated in artificial seawater. After 24 h incubation at 25 °C, the nauplii were transferred to microplates containing seawater and 5–100 µg GAE/mL of each extract. The experiment included solvent controls (dimethyl sulfoxide, DMSO) without extract and a positive control of potassium dichromate (10–40 µg/mL). Each treatment was performed in triplicate with 10 nauplii per well. After 24 h at 25 °C of incubation, mortality was recorded and the corresponding percentage was calculated as (number of deaths/initial number of nauplii) × 100.

### 3.8. Total and Relative Carbon Footprint

The total carbon footprint of each extraction method of TPC was estimated considering the electrical energy consumed during the process and the produced carbon dioxide for the regional energy matrix of Argentina (0.387 kg de CO_2_/kWh) [55,56].

The energy consumption (E) was calculated:

E = P × T

P is the nominal power of the equipment used (in kilowatts, kW); T is the effective extraction time (in hours). The total carbon footprint (CF) was calculated by multiplying the energy consumption by the carbon dioxide emission factor (F) for the regional energy matrix:

CF = E × F (kg CO_2_/kWh).

The relative carbon footprint (RCF) was calculated as amount of CO_2_ emissions per milligram of TPC extracted:

RCF = CF/mg TPC

RCF allowed for an objective evaluation of the sustainability of the analyzed methods, considering the environmental footprint per unit of functional compound obtained.

### 3.9. Statistical Analysis

The free trial of Design-Expert software version 13.0.0 was used to perform the optimization experiment and modeling. R Studio Team (2020) software was used for statistical analysis. A one-way analysis of variance (ANOVA) test was used to assess the significance of the influence of independent variables and interactions, followed by the Tukey post hoc test. All results were expressed as the mean ± standard deviation. At *p*-value ≤ 0.05, differences between samples were considered statistically significant.

## 4. Conclusions

It was demonstrated that ultrasound-assisted extraction (UAE) is an efficient technique for obtaining antioxidant polyphenol-rich extracts from *Cannabis sativa* leaf powder. The optimal extraction conditions were determined using response surface methodology (46% ethanol and a 1:10 *w*/*v* solid-to-solvent ratio), with an optimal extraction time of 6 min, after which no significant increase in yield was observed. Under these conditions, the maximum yields of total phenolic compounds and total flavonoids obtained experimentally were 1746.83 µg GAE/mL and 858.41 µg QE/mL, respectively, in agreement with the model predictions. The extract produced under these optimal conditions was enriched in flavonoids, such as luteolin, rutin, kaempferol, diosmetin, apigenin, and C-glycosides derived from apigenin, and exhibited high antioxidant capacity. Notably, extraction at lower ethanol concentrations favored the recovery of phenolic compounds, particularly flavonoids, while avoiding the co-extraction of psychoactive THC-type cannabinoids. According to Argentine regulations, these extracts could be suitable for the development of medicinal products. From an environmental perspective, the process showed relatively low energy consumption at laboratory scale (0.329 kWh per batch), corresponding to a total carbon footprint of 0.12 kg CO_2_ per extraction, and a normalized value of 0.0028 kg CO_2_/mg GAE. These results highlight the potential of UAE as an energy-efficient technique for the recovery of antioxidant compounds from agro-industrial residues. However, some limitations must be acknowledged. The results are based on laboratory-scale experiments and therefore may not fully represent the environmental and operational performance at larger scales. Energetic efficiency and carbon footprint may change significantly during scale-up due to differences in ultrasonic-energy distribution, mass transfer limitations, reactor geometry, and process intensification strategies. Additionally, the carbon footprint associated with raw material production, especially cultivation conditions, was not included and could represent a major contribution to the overall life cycle impact. Therefore, future work should focus on pilot-scale validation and life cycle assessment (LCA) studies to better understand the environmental implications of scaling up the process. The use of continuous-flow ultrasonic systems, optimization of energy input, and integration with green extraction strategies should also be explored to enhance process sustainability. Overall, this research provides novel evidence supporting the valorization of *C. sativa* leaf powder, a byproduct of the Argentine cannabis industry, as a sustainable source of antioxidant compounds, contributing to circular economy approaches.

## Figures and Tables

**Table 1:** Particle size distribution of *Cannabis sativa* leaf powder. The percentages indicate the proportion of material retained on each sieve after five minutes of shaking. The highest retention was observed with sieve no. 100 (149 µm). Three replicates were performed.

Sieve No.	Opening (µm)	Powder (%)
20	840	0.93 ± 1.03
35	500	4.52 ± 3.16
100	149	29.80 ± 4.07
140	105	25.24 ± 2.80
200	74	15.73 ± 2.00
Collector	0	16.69 ± 3.75

**Table 2:** Compounds identified in each extract of *Cannabis sativa* leaves.

Peak No.	Retention Time (min)	Identified Compounds	Distilled Water	Ethanol 40%	Ethanol 60%	Ethanol 96%
1	2.705	NI	X	X	X	X
2	3.098	NI		X	X	X
3	5.583	NI		X	X	
4	7.410	Kaempferol		X	X	
5	8.388	Diosmetin		X	X	
6	9.028	Vitexin		X	X	
7	9.914	Isovitexin		X	X	
8	10.004	Rutin		X	X	
9	12.097	NI		X	X	
10	13.231	Luteolin	X	X	X	
11	13.544	Apigenin	X	X	X	
12	19.319	CBD-A			X	X
13	19.924	CBD			X	X
14	21.724	THC			X	X
15	22.858	THC-A			X	X
16	23.585	NI			X	X

An ‘X’ denotes the presence of the compound in the respective extract. NI: Not identified.

**Table 3:** Response surface methodology of a three-variable three-level unbalanced full factorial design to model the extraction of total phenolic compounds (TPC) and total flavonoids (TF) of *Cannabis sativa* extracts using UAE.

Run No.	Factors			Responses ^1^	
	S/L Ratio (g/mL)	Time (min)	Solvent (%)	TPC (µg GAE/mL)	TF (µg QE/mL)
1	1:10	1	0	1017.66	560.2
2	1:20	1	0	743.93	432.21
3	1:40	1	0	548.69	112.36
4	1:10	6	0	1454	689.7
5	1:20	6	0	808.5	202.24
6	1:40	6	0	478.2	145.32
7	1:10	12	0	1405.4	419.9
8	1:20	12	0	753.5	244.95
9	1:40	12	0	517.8	229.21
10	1:10	1	60	1559.97	1075
11	1:20	1	60	969.09	808.98
12	1:40	1	60	675.39	327.34
13	1:10	6	60	1565.1	936.5
14	1:20	6	60	896.8	730.33
15	1:40	6	60	550.1	342.32
16	1:10	12	60	1598.2	1110
17	1:20	12	60	963.86	594.76
18	1:40	12	96	527.6	277.15
19	1:10	1	96	984.9	193.37
20	1:20	1	96	488.3	75.66
21	1:40	1	96	247.9	98.13
22	1:10	6	96	904.7	542.1
23	1:20	6	96	507.02	260.68
24	1:40	6	96	241.1	117.6
25	1:10	12	96	1068.4	520.8
26	1:20	12	96	527.66	86.15
27	1:40	12	96	254.2	119.1
28	1:20	6	60	805.7	618.5
29	1:20	6	60	807.1	598.95
30	1:20	6	60	810.3	631.76
31	1:20	6	60	828.4	611.52

^1^ Values are presented as mean (*n* = 3). GAE: Gallic acid equivalents; QE: quercetin equivalents; TPC: total phenolic compounds; TF: total flavonoids.

**Table 4:** Analysis of variance (ANOVA) for the fitted regression model of total phenolic compounds (TPC) and flavonoids (TF) from *Cannabis sativa* using ultrasound-assisted extraction (UAE).

TPC						
Source	Sum of Squares	df^1^	Mean Square	F-Value	*p*-Value	
Model	1321.68	6	220.28	267.23	<0.0001 ***	significant
A—Solid-to-Liquid Ratio	1004.27	1	1004.27	1218.34	<0.0001 ***	
B—Time	0.1095	1	0.1095	0.1329	0.7188	
C—Solvent	162.70	1	162.70	197.38	<0.0001 ***	
A^2^	134.41	1	134.41	163.06	<0.0001 ***	
B^2^	5.02	1	5.02	6.09	0.0215 *	
C^2^	214.23	1	214.23	259.89	<0.0001 ***	
Residual	18.96	23	0.8243			
Lack of Fit	17.21	19	0.9056	2.07	0.2524	not significant
Pure Error	1.75	4	0.4379			
Cor Total	1340.64	29				
TF						
Source	Sum of Squares	df^1^	Mean Square	F-value	*p*-value	
Model	2.27 × 10^9^	4	5.66 × 10^8^	49.21	<0.0001 ***	significant
A—Solid-to-Liquid Ratio	1.12 × 10^9^	1	1.12 × 10^9^	96.99	<0.0001 ***	
C—Solvent	23,305.31	1	23,305.31	2.03	0.1671	
A^2^	1.81 × 10^8^	1	1.81 × 10^8^	15.72	0.0005 **	
C^2^	1.05 × 10^9^	1	1.05 × 10^9^	91.19	<0.0001 ***	
Residual	2.88 × 10^8^	25	11,508.55			
Lack of Fit	2.77 × 10^8^	21	13,168.75	4.72	0.0712	not significant
Pure Error	11,169.88	4	2792.47			

* Means significant at *p* ≤ 0.05; ** means highly significant at *p* ≤ 0.01; *** means extremely significant at *p* ≤ 0.0001; df^1^: degrees of freedom.

**Table 5:** Comparison between predicted and experimental yields of total phenolic compounds (TPC) and flavonoids (TF) from *Cannabis sativa* under optimal conditions using ultrasound-assisted extraction (UAE), considering two-sided 95% prediction interval (PI).

Analysis	Predicted Mean	Std Dev	N	SE Pred	95% PI Low	Data Mean	95% PI High
TPC (µg GAE/mL)	1697.9	74.8128	3	N/A	1574.91	1746.83	1823.81
TF (µg QE/mL)	1002.35	107.278	3	779.647	841.775	858.407	1162.92

GAE: Gallic acid equivalents; QE: quercetin equivalents; N/A: not applicable.

**Table 6:** Factors and corresponding levels used in the unbalanced full factorial experimental design.

Factors	Symbol	Levels		
Solid-to-liquid ratio (g/mL)	A	1:10	1:20	1:40
Time (min)	B	1	6	12
Solvent (%)	C	0	60	96

## Data Availability

The original contributions presented in this study are included in the article. Further inquiries can be directed to the corresponding author.
